# Preseason Y Balance Test Scores are not Associated with Noncontact Time-Loss Lower Quadrant Injury in Male Collegiate Basketball Players

**DOI:** 10.3390/sports7010004

**Published:** 2018-12-24

**Authors:** Jason Brumitt, Kyle Nelson, Duane Duey, Matthew Jeppson, Luke Hammer

**Affiliations:** 1 George Fox University, 414 N. Meridian St, Newberg, OR 97132, USA; mjeppson15@georgefox.edu (M.J.); lhammer11@georgefox.edu (L.H.); 2 Concordia University, Portland, OR 97132, USA; kynelson@cu-portland.edu; 3 Linfield College, McMinnville, OR 97132, USA; dduey@linfield.edu

**Keywords:** balance, epidemiology, functional performance test, injury screening, preseason

## Abstract

The Y-Balance Test-Lower Quarter has shown promise as a screening tool for identifying athletes at risk of injury. Subsequent studies, utilizing heterogeneous populations or different operational definitions of injury, have presented equivocal findings. Therefore, studies evaluating the efficacy of the Y-Balance Test to discriminate injury risk in a homogeneous population is warranted. One-hundred sixty-nine male (mean age 19.9 ± 1.5 y) collegiate basketball players were recruited during 2 consecutive seasons (2016–2017/2017–2018). Athletes completed the Y-Balance testing protocol at the start of each preseason. Athletic trainers tracked noncontact time-loss lower quadrant injuries over the course of the season. Receiver operator characteristic curves failed to identify cutoff scores; therefore, previously reported cutoff scores were utilized when calculating relative risk. There was no association between preseason Y-Balance Test scores and noncontact time-loss lower back or lower extremity injury in a population of male collegiate basketball players. This study adds to a growing body of evidence that demonstrates no relationship between preseason Y-Balance Test scores and subsequent injury.

## 1. Introduction 

The Y-Balance Test–Lower Quarter (YBT-LQ) is a functional performance test utilized to evaluate dynamic balance during clinical rehabilitation [1,2,3,4,5,6]. The YBT-LQ has also been used to identify athletes who may be at a greater risk of injury [7,8,9,10,11,12,13]. If a functional performance test, like the YBT-LQ, can identify athletes at risk of injury, then clinicians could prescribe an injury prevention training program to address an athlete’s deficits.

The YBT-LQ had shown early promise as a preseason screening tool to discriminate injury risk in athletic populations. Plisky et al. [14], using the star excursion balance test (SEBT; a precursor to the YBT-LQ) [15], evaluated preseason dynamic balance in high school basketball (BB) players. High school BB players (both sexes) with asymmetrical anterior reach scores greater than 4 cm were more than twice as likely to experience a time-loss lower extremity injury, and female BB players with lower composite scores were 6.5 times more likely to be injured [14]. Butler et al [7], utilizing the YBT-LQ also reported that a lower composite score (albeit a different one from Plisky et al. [14]) was associated with a three-fold risk of a noncontact lower extremity injury in collegiate football players [7].

Since these initial reports, several prospective cohort studies have evaluated the ability of the YBT-LQ to discriminate injury risk in athletic populations [8,9,10,11,12,13]. However, the equivocal findings from these studies challenge a clinician’s ability to interpret YBT-LQ scores collected during a screening clinic. Potential reasons associated with the equivocal findings may be due to one or more factors: the population studied (e.g., heterogeneous or homogeneous populations), the operational definition of injury utilized, test performance modification, and the injury surveillance period [7,8,9,10,11,12,13]. Therefore, additional studies utilizing a homogeneous population and a standardized methodology are warranted to determine the efficacy of this test as preseason screening tool.

One population of athletes whose YBT-LQ scores have not been prospectively evaluated independent of other athletes are male collegiate basketball players. The purpose of this study was to evaluate the ability of the YBT-LQ to discriminate injury risk in a population of male collegiate BB players. It was hypothesized that male collegiate BB players who presented with either a) a lower composite score, or b) an asymmetrical reach measure, would have a significantly greater risk of sustaining a noncontact time-loss injury to the lower back or to lower extremities.

## 2. Methods

### 2.1. Participants

Male collegiate BB players were recruited from National Collegiate Athletic Association (NCAA) Division II (D II), NCAA Division III (D III), National Association of Intercollegiate Athletics (NAIA), and community college teams from the Portland, Oregon region. Athletes were recruited in a 2-step process. First, the primary investigator contacted each team’s head coach and athletic trainer (ATC) by phone or email to seek their participation. Next, if the coach and ATC agreed to participate, the primary investigator emailed team members, inviting them to a scheduled testing session. A total of 169 BB players were tested at the start of the 2016–2017 and 2017–2018 preseasons: [2016: 1 D II team (n = 14); 3 D III teams (n = 45); 2 NAIA teams (n = 18); 1 community college team (n = 17); 2017: 1 D II team (n = 16); 3 D III teams (n = 36); 1 NAIA team (n = 10); 1 community college team (n = 13)]. Of the 169 athletes, a total of 35 participated in the study both years. All athletes were evaluated at the start of each preseason. Informed consent was obtained from each participant prior to testing. The Institutional Review Board of George Fox University (Newberg, OR) approved this study.

### 2.2. YBT-LQ Testing Protocol

Each athlete, prior to testing, completed a 5-minute dynamic warm-up consisting of the following movements: forward lunging, backward lunging, heel walking, tip toe walking, marching, and toy soldiers. Next, athletes were provided with YBT-LQ test performance instruction and completed six warm-up trials [15]. Test performance was conducted as described by Plisky et al. [15]. Subjects were instructed to stand barefoot on the weightbearing platform with one foot positioned behind the red indicator line. Next, the athlete would reach into one of three directions (anterior, posteromedial and posterolateral) using their non-weight-bearing lower extremity to slide the moveable platform. Three reach trials were performed; first in the anterior direction starting on the right side (e.g., right lower extremity in stance with left lower extremity non-weight-bearing) followed by three trials on the left [7,15]. Once the anterior trials were completed, the athlete performed three trials bilaterally (right followed by left) into the posteromedial, and lastly, the posterolateral directions [7,15]. Any failed reach trial was repeated until correctly performed [7,15]. An investigator, either the primary investigator or a graduate research assistant, measured each successful trial.

YBT-LQ reach scores were normalized as a percentage of limb length (see statistical analysis section). Limb length was measured from the anterior superior iliac spine to the distal aspect of the medial malleolus, using a cloth measuring tape, after completing the YBT-LQ trials [15]. The intra- (0.85–0.91) [15] and inter-rater reliability (0.85–0.93 [16] and 0.99–1.00 [15]) for the YBT-LQ is excellent.

### 2.3. Injury Surveillance

The following information was collected by ATCs for each injured athlete: location, injury diagnosis, and the mechanism of injury (e.g., noncontact or contact). The operational definition of an injury for this study was any noncontact musculoskeletal injury to the lower back or lower extremities that occurred during a practice or game that resulted in the athlete being removed from that day’s event or that prevented the athlete from participating in the next scheduled event [17,18].

### 2.4. Statistical Analysis

Prior prospective cohort studies that have reported significant associations between YBT-LQ scores and future injury risk in homogeneous populations utilized sample sizes ranging from 59 subjects (male collegiate football players) [7] to 74 subjects (professional and amateur soccer players) [8]. A sample size estimation (with power of 0.80, alpha level of 0.05, and estimating a 25% injury rate) of 118 subjects was necessary to determine statistically-significant associations for this study.

Asymmetrical reach measures and composite reach scores were calculated for subsequent risk analysis. Mean individual reach scores were normalized as a percentage of limb length [(reach distance/limb length) × 100]. Next, side-to-side differences were calculated between lower extremities per each reach direction. The composite reach score was calculated using this formula: [(mean Anterior + mean Posteromedial + mean Posterolateral)/(limb length × 3)] × 100 [7,15].

Receiver operator characteristic (ROC) curves were constructed for each reach asymmetry and each set of composite scores. Each ROC curve failed to identify a cutoff score that would maximize sensitivity and specificity; therefore, previously reported cutoff scores for reach asymmetry [8,11,14] and composite scores [7,14] were utilized. Athletes were categorized as “at risk” if their side-to-side reach asymmetry was >4 cm in any one of the reach directions or if their composite score was <89.6% [8] or <94% [14]. Cutoff scores were used to calculate the relative risk (RR) of a noncontact time-loss injury to the lower back or to lower extremities (i.e., “all injuries”) or a noncontact time-loss ankle sprain injury. Statistical analyses were performed using SPSS 24.0 (Chicago, IL) for all calculations.

## 3. Results

Forty-three athletes (25.4 percent of the sample) experienced a noncontact time-loss lower quadrant injury during the study. Table 1 presents the relative risk of injury per YBT-LQ categories. There were no significant associations found between preseason YBT-LQ scores and injury.

## 4. Discussion

Preseason YBT-LQ scores were not associated with a noncontact time-loss lower quadrant (low back or lower extremity) injury in a population of male collegiate BB players. This prospective cohort study adds to the growing body of research demonstrating that no relationship exists between preseason YBT-LQ and injury in athletic populations.

As previously mentioned, the YBT-LQ was inspired by the results from astudy that valuated the ability of the SEBT to discriminate injury risk in high school BB players [14]. Subsequent prospective cohort studies evaluating the ability of the YBT-LQ to discriminate injury have been equivocal; this is perhaps due to different methodological considerations [7,8,9,10,11,12,13]. 

First, the operational definition of injury utilized in a study may account for significant findings. Most prospective cohort studies investigating the ability of a functional performance test to discriminate injury risk only count noncontact time-loss injuries [7,8,10,12,13,14,17,19,20]. Two studies that reported a significant association between test performance and injury included either non-time-loss injuries or injuries resulting from a contact mechanism in the analysis of risk. For example, Smith et al. [11] assessed preseason YBT-LQ performance in a heterogeneous population consisting of 184 Division I (D I) athletes (males = 102) representing nine sports. Athletes who had an anterior reach asymmetry > 4 cm were two times more likely to experience a noncontact injury; however, both time-loss and non-time-loss injuries were included in this study’s risk analysis [11]. The inclusion of non-time-loss injuries when assessing risk adds a degree of subjectivity regarding the severity of those “injuries”, and potentially changes the significance of the association. Hartley et al. [9] prospectively evaluated the ability of the YBT-LQ to discriminate ankle sprain injury risk in a heterogeneous population of 551 collegiate athletes. The authors reported a significant association between anterior reach (<54.4% of limb length in anterior direction) performance and ankle sprain; however, they included injuries resulting from both noncontact and contact mechanisms [9]. There are two limitations associated with including injuries resulting from contact mechanisms when calculating risk. The inclusion of contact related injuries potentially changes the significance of the relative risk calculation associated with that sample. In addition, the goal of a preseason screen is to identify at risk athletes, and then intervene with a training program to reduce the risk of injury. Upwards of 50 percent of ankle sprains during BB are a result of player contact [21,22,23]. It is highly unlikely that a training program could reduce injury risk associated with player-to-player contact.

Second, the heterogeneity of a study’s population may influence a study’s findings. Several studies utilizing heterogeneous populations (D I athletes [10,13], D II women’s BB/women’s volleyball [12] and D III athletes [unpublished data, 2019] [24]) have reported no association between preseason scores and sports injury. Potential reasons for the finding of no association between test scores and injury may be due to differences in injury risk between sports or differences in normalized reach performance between genders [16,25,26,27]. Contrary to the findings associated with the aforementioned studies, two studies utilizing homogeneous populations (male collegiate football players = 59 [7] and male professional and amateur soccer players = 74 [8]) have reported significant relationships between either composite score [7] or reach asymmetry [8] and injury. However, these findings have not been validated in a second sample. To our knowledge this current study evaluated the largest homogeneous sample (n = 169) finding no relationship between scores and injury.

Third, the injury surveillance period may also influence a study’s findings. Typically, athletes are prospectively observed over the course of a season [7,8,10,12,13,14,17,19,20]. However, Hartley et al. [9] followed subjects for up to a two-year period, and it appears that the athletes were only tested at the start of the first year of the study and not at the start of each sport season. It is possible that one’s year-to-year performance changed in response to prior injury or training habits.

Finally, modifications in test performance may account for differences in findings between studies. For example, Hartley et al. [9] deviated from test performance (as defined by Plisky et al. [15]) by having athletes maintain their hands on their hip during test performance.

The aforementioned methodological concerns challenge a clinician’s ability to interpret the significance of test scores when evaluating athletes during a preseason screening clinic. A reliable screening test should consistently discriminate injury risk in athletic populations based on similar variables (e.g., composite score or anterior reach asymmetry). To date, only two studies evaluating test performance in homogeneous populations have reported significant associations; however, one study reported an association based on composite score and the other based on posteromedial reach asymmetry [7,8]. As previously mentioned, neither of these studies have been validated in a follow-up sample. It is not uncommon for an initial study to report a significant relationship between test performance and injury only to be followed by a validation study that did not find an association [10,11,13,19,20].

It should be noted that studies utilizing the SEBT, the precursor to the YBT-LQ, have demonstrated significant associations between preseason test performance and injury [14,28,29,30]. Athletes demonstrate different postural-control strategies when performing the SEBT when compared to the YBT-LQ, which is reflected in their anterior reach performance [31,32]. It is possible that the SEBT could be a better a screening tool than the YBT-LQ; however, this would require future research to determine its efficacy in a male collegiate BB population.

The strengths and weaknesses of this study should be addressed. The strengths of this study include the study’s sample size, the prospective collection of YBT-LQ measures, the operational definition of an injury, the collection of injury data by certified athletic trainers, and the weekly monitoring of injury information by the primary investigator. A potential weakness associated with this study is that the sample size was likely not large enough to assess injury risk per specific diagnoses. For example, Hartley et al. [9] recruited over 500 athletes to analyze ankle sprain risk based on YBT-LQ scores. A future study using either the SEBT or YBT-LQ would need to consist of a larger sample size.

## 5. Conclusion

The YBT-LQ did not discriminate injury risk in male collegiate basketball players. This study adds to a growing body of literature that has found no relationship between YBT-LQ test performance and future sports injury.

## Figures and Tables

**Table 1:** Relative Risk of Noncontact Time-Loss Injury per YBT-LQ Variables.

Variable	N at Risk	All Injuries ^‡^ Counts and (%) per Category	Relative Risk (95% CI)	Ankle Sprain Injury Counts and (%) per Category	Relative Risk (95% CI)
Anterior Reach Asymmetry					
4 cm or less	98	24 (24.5)	1.0 (Reference)	6 (6.1)	1.0 (Reference)
>4 cm	71	19 (26.8)	1.1 (0.7, 1.8)	5 (7.0)	1.2 (0.4, 3.6)
Posteromedial Reach Asymmetry					
4 cm or less	91	25 (27.5)	1.0 (Reference)	6 (6.6)	1.0 (Reference)
>4 cm	78	18 (23.1)	0.8 (0.5, 1.4)	5 (6.4)	1.0 (0.3, 3.1)
Posterolateral Reach Asymmetry					
4 cm or less	56	16 (28.6)	1.0 (Reference)	4 (7.1)	1.0 (Reference)
>4 cm	113	27 (23.9)	0.8 (0.5, 1.4)	7 (6.2)	0.9 (0.3, 2.8)
Right Composite Score					
94% or greater	51	13 (25.5)	1.0 (Reference)	4 (7.8)	1.0 (Reference)
<94%	118	30 (25.4)	1.0 (0.6, 1.7)	7 (5.9)	0.8 (0.2, 2.5)
89.6% or greater	86	20 (23.3)	1.0 (Reference)	4 (4.7)	1.0 (Reference)
<89.6%	83	23 (27.7)	1.2 (0.7, 2.0)	7 (8.4)	1.8 (0.6, 6.0)
Left Composite Score					
94% or greater	55	15 (27.3)	1.0 (Reference)	2 (3.6)	1.0 (Reference)
<94%	114	28 (24.6)	0.9 (0.5, 1.5)	9 (7.9)	2.2 (0.5, 9.7)
89.6% or greater	92	25 (27.2)	1.0 (Reference)	6 (6.5)	1.0 (Reference)
<89.6%	77	18 (23.4)	0.9 (0.5, 1.5)	5 (6.5)	1.0 (0.3, 3.1)

CI = confidence interval. ^‡^ = noncontact time-loss injury to the lower back or to the lower extremities.
